# A Putative Hsa-miR-582-5p–CD81 Relationship Identified by Integrative Transcriptomic Analysis in Osteosarcoma

**DOI:** 10.3390/ijms27031558

**Published:** 2026-02-05

**Authors:** Ju-Fang Liu, Tsung-Ming Chang, Chi-Jen Chang, Peng Chen, Ying-Sui Sun

**Affiliations:** 1 School of Oral Hygiene, College of Oral Medicine, Taipei Medical University, Taipei 11031, Taiwan; jufangliu@tmu.edu.tw; 2 Translational Medicine Center, Shin Kong Wu Ho-Su Memorial Hospital, Taipei 111045, Taiwan; 3 Department of Medical Research, China Medical University Hospital, China Medical University, Taichung 40402, Taiwan; 4 School of Dental Technology, College of Oral Medicine, Taipei Medical University, Taipei 11031, Taiwan; a03441@tmu.edu.tw; 5 School of Medicine, Fu Jen Catholic University, New Taipei City 24205, Taiwan; m002008@ms.skh.org.tw; 6 Division of Pediatric Surgery, Shin Kong Wu Ho-Su Memorial Hospital, Taipei 111045, Taiwan; 7 Liaison Center for Innovative Dentistry, Graduate School of Dentistry, Tohoku University, Sendai 980-8575, Japan; peng.chen.b7@tohoku.ac.jp

**Keywords:** osteosarcoma, biomarker, *CD81*, hsa-miR-582-5p, integrative bioinformatics, target prediction, prognosis

## Abstract

Osteosarcoma (OS) is the most common primary malignant bone tumor in adolescents, and outcomes for metastatic disease have remained poor, highlighting the need for molecular biomarkers. We integrated three Gene Expression Omnibus (GEO) mRNA expression datasets (GSE12865, GSE14359, and GSE246405) to identify differentially expressed genes (DEGs) between OS and non-malignant bone-related controls. Overlapping DEGs were used to build a protein–protein interaction network, and hub genes were prioritized using multiple network topology algorithms. Prognostic associations were evaluated using the R2 Genomics Platform. Putative upstream miRNAs targeting the top candidate were obtained from prediction databases and intersected with dysregulated circulating miRNAs from GSE65071 (localized OS plasma vs. healthy controls). Functional enrichment analyses (Gene Ontology (GO), Kyoto Encyclopedia of Genes and Genomes (KEGG), and cancer hallmarks) were performed to contextualize the candidate signature. We identified 107 overlapping DEGs and prioritized eight hub genes. *CD81* was significantly associated with overall survival (Bonferroni-adjusted *p* = 0.043) and showed reduced expression in OS tissues and cell line models. hsa-miR-582-5p was nominated as a candidate miRNA predicted to target *CD81* and was upregulated in OS plasma. Enrichment results linked the signature to angiogenesis, extracellular matrix remodeling, focal adhesion, and metastasis-associated signatures. These findings support *CD81* as a candidate prognostic biomarker and nominate a putative hsa-miR-582-5p–*CD81* relationship for future validation.

## 1. Introduction

Osteosarcoma (OS) is the most common primary malignant bone tumor in children and adolescents, with incidence peaks during adolescence and later adulthood [1]. Despite advances in chemotherapy and limb-sparing surgery, overall survival has plateaued since the 1980s, especially in metastatic or relapsed cases [2]. Historically, osteosarcoma outcomes improved substantially after the introduction of multi-agent chemotherapy; however, survival gains have largely plateaued since the late 1980s to1990s. Contemporary series report ~60–70% 5-year survival for localized disease treated with standard multimodality therapy, whereas outcomes for patients presenting with metastasis remain poor (approximately 10–40% survival), with pulmonary micrometastatic disease being a major driver of treatment failure. These limitations highlight the need for improved risk stratification and molecular biomarkers to better understand aggressive biology and guide future translational studies [2]. Conventional prognostic factors, including tumor size, metastatic status, and surgical completeness, remain clinically relevant, yet metastatic disease continues to predict poor outcomes [3,4]. These clinical challenges underscore the critical need for reliable molecular biomarkers capable of stratifying patient risk and informing future translational studies. The development of biomarkers for early detection, disease monitoring, and prognostication represents a promising avenue to improve survival outcomes in osteosarcoma patients.

Cluster of Differentiation 81 (CD81), a member of the tetraspanin family, has been implicated in cancer progression and metastatic dissemination. As a molecular organizer of tetraspanin-enriched microdomains (TEMs), CD81 serves as a dynamic scaffold that orchestrates protein–protein interactions essential for cell adhesion, migration, and malignancy-associated signaling [5]. Beyond its canonical role in organizing membrane architecture, CD81 has been reported to modulate oncogenic signaling pathways, including focal adhesion kinase (FAK), phosphoinositide 3-kinase (PI3K)/protein kinase B (Akt), and extracellular signal-regulated kinase (ERK), in a context-dependent manner, potentially influencing cytoskeletal remodeling and cell motility [6].

Accumulating evidence demonstrates that aberrant *CD81* expression promotes tumor growth and metastatic progression across multiple malignancies. In melanoma, osteosarcoma, and triple-negative breast cancer, elevated *CD81* correlates with enhanced invasive capacity and poor clinical outcomes [7,8,9]. Conversely, *CD81* knockout studies have demonstrated reduced tumor growth, attenuated cell migration, and diminished spontaneous metastasis, underscoring a tumor-intrinsic role of *CD81* in cancer progression [8]. Collectively, prior studies suggest that *CD81* may be relevant to osteosarcoma biology and highlight its potential as a biomarker candidate for further systematic evaluation.

MicroRNAs (miRNAs) are small, non-coding RNAs approximately 18–25 nucleotides in length that regulate gene expression post-transcriptionally by binding to target mRNAs, leading to degradation or translational repression. They play essential roles in diverse biological processes, including cell proliferation, differentiation, apoptosis, and stress responses. Dysregulated miRNA expression is a hallmark of cancer and contributes to tumor initiation, progression, metastasis, and therapy resistance by modulating oncogenes and tumor suppressors [10,11,12]. Aberrant miRNA networks have been implicated in multiple malignancies—such as hepatocellular carcinoma, lung cancer, and sarcomas—through the regulation of signaling pathways including PI3K/Akt, Mitogen-activated protein kinase (MAPK), and Signal Transducer and Activator of Transcription 3 (STAT3). In OS, numerous studies have identified distinct miRNA expression profiles that differentiate tumor from normal bone tissue, suggesting potential roles as diagnostic and prognostic biomarkers [13]. Specific miRNAs have been shown to regulate processes such as angiogenesis, invasion, metastasis, and chemoresistance. Collectively, these findings underscore miRNAs as pivotal regulators of osteosarcoma biology and promising non-invasive biomarker candidates and a potential source of testable therapeutic hypotheses.

In this study, we applied a multi-dataset integrative bioinformatic framework to systematically investigate molecular networks relevant to osteosarcoma progression. Our emphasis on cell adhesion- and microenvironment-related processes was data-driven: across independent osteosarcoma transcriptomic datasets, consistently dysregulated genes converged on extracellular matrix remodeling, focal adhesion-associated signaling, and other invasion-related programs, which are central to metastatic dissemination. Accordingly, we integrated publicly available mRNA expression datasets to identify reproducibly altered genes, constructed protein–protein interaction networks, and prioritized candidate hub genes using complementary network-topology algorithms, followed by prognostic association assessment in an independent survival cohort. To explore a putative post-transcriptional regulatory layer, we further combined miRNA target prediction with circulating miRNA profiling to nominate testable miRNA–mRNA relationships. Collectively, this study aims to prioritize candidate prognostic biomarkers and generate hypothesis-driven directions for subsequent validation in clinical specimens and functional assays.

## 2. Results

### 2.1. Identification of Differentially Expressed Genes (DEGs) in Osteosarcoma

**Figure 1:**
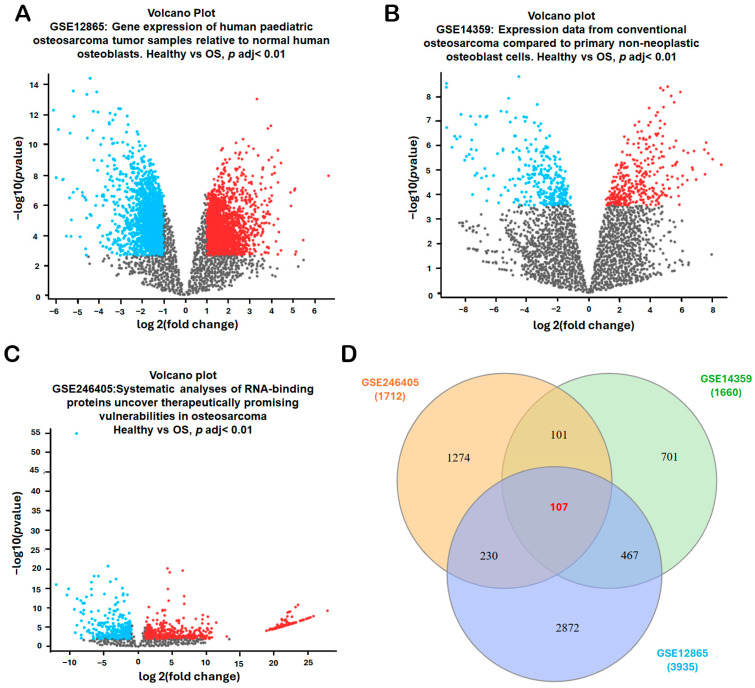
Identification of Differentially Expressed Genes (DEGs) in Osteosarcoma. (**A**–**C**) Volcano plots were generated for three GEO datasets (GSE12865, GSE14359, and GSE246405) to visualize the distribution of differentially expressed genes (DEGs) between osteosarcoma and non-malignant controls. Data were normalized, and DEGs were filtered using an adj. *p*-value < 0.01 and |log_2_FC| > 1.0. The volcano plots highlight significantly upregulated genes (red), downregulated genes (blue), and non-significant genes (gray). Each dot represents one gene; the x-axis indicates log2(fold change) and the y-axis indicates −log10(adjusted *p*-value). (**D**) Overlapping DEGs among the three datasets were identified using the InteractiVenn platform to obtain consistently dysregulated genes for subsequent analyses. In the Venn diagram (**D**), each colored circle corresponds to one dataset (GSE12865, GSE14359, and GSE246405), and the numbers indicate the counts of DEGs in each region.

To identify robust differentially expressed genes (DEGs) associated with osteosarcoma diagnosis and disease progression, three publicly available microarray datasets—GSE12865, GSE14359, and GSE246405—were systematically analyzed from the Gene Expression Omnibus (GEO) database (National Center for Biotechnology Information, Bethesda, MD, USA; https://www.ncbi.nlm.nih.gov/geo/; accessed 2 November 2025). Each dataset included transcriptomic profiles comparing osteosarcoma samples with non-malignant bone-related controls (normal osteoblast/bone controls depending on the dataset). The volcano plots for each dataset illustrated the distribution of DEGs, highlighting significantly upregulated genes (red), downregulated genes (blue), and non-significant genes (gray) in osteosarcoma samples (Figure 1A–C). The combined datasets encompassed 18 osteosarcoma samples and 4 non-malignant controls—specifically, GSE12865 (tumor = 6, osteoblast control = 1; technical duplicates), GSE14359 (conventional OS = 5, osteoblast controls (HOBc) = 1; technical duplicates for each sample), and GSE246405 (OS models = 4; osteoblast control models = 1; one OS model lacked a replicate) (Table 1). To identify consistently dysregulated genes across these independent datasets, a Venn diagram intersection analysis was conducted. The comparison of DEGs from GSE12865 (3935 DEGs), GSE14359 (1660 DEGs), and GSE246405 (1712 DEGs) revealed 107 overlapping DEGs (Figure 1D). These shared genes represent a core transcriptional signature of osteosarcoma and were prioritized for subsequent functional enrichment, pathway analysis, and biomarker validation.

### 2.2. Functional Enrichment Analysis of Common DEGs

**Figure 2:**
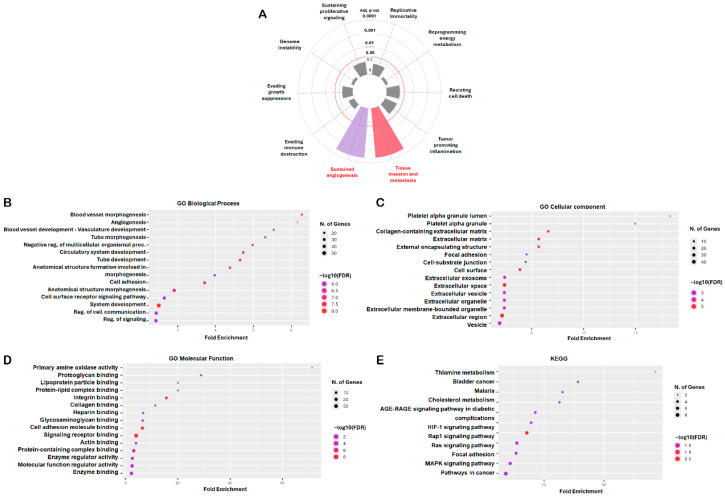
Functional Enrichment Analysis of DEGs. The Cancer Hallmarks Analytics Tool and ShinyGO v0.82 platforms were used to analyze the biological significance of 107 shared DEGs. (**A**) Hallmark pathway analysis. Gray sectors indicate non-significant cancer hallmark terms. The concentric circles represent adjusted *p*-value thresholds from inner to outer: 1, 0.1, 0.05, 0.01, 0.001, and 0.0001. The red dashed circle marks adjusted *p*-value = 0.05, whereas the other circles are shown as gray solid lines. (**B**–**E**) The Gene Ontology (GO) and KEGG pathway analyses classified genes into biological process (BP), cellular component (CC), molecular function (MF), and signaling pathway categories. For the dot plots (**B**–**E**), dot size represents the number of genes, and dot color represents −log_10_(FDR); the x-axis indicates fold enrichment and the y-axis lists enriched terms. Statistical significance was set at adj. *p* < 0.05 and FDR < 0.05.

To elucidate the biological significance and molecular mechanisms underlying the 107 consistently dysregulated genes identified across the three osteosarcoma datasets, we conducted comprehensive enrichment analyses using both the Cancer Hallmarks Analytics Tool (Semmelweis University, Budapest, Hungary; https://cancerhallmarks.com; accessed 3 November 2025) and the ShinyGO v0.82 platform (South Dakota State University, Brookings, SD, USA; http://bioinformatics.sdstate.edu/go/; accessed 4 November 2025). Analysis through the Cancer Hallmarks platform revealed that these DEGs were significantly associated with hallmark oncogenic processes, including sustained angiogenesis, tissue invasion, and metastasis (Figure 2A). Complementary Gene Ontology (GO) analysis using ShinyGO v0.82 further highlighted extensive functional enrichment across multiple biological categories relevant to osteosarcoma pathology. In the biological process (BP) category, enriched terms primarily involved blood vessel morphogenesis, angiogenesis, vasculature development, tube morphogenesis, cell adhesion, and regulation of signaling pathways, reflecting the strong involvement of these genes in vascular remodeling and cellular communication (Figure 2B). The cellular component (CC) category indicated that these DEGs were predominantly localized to extracellular and adhesion-related regions, including the platelet alpha granule, collagen-containing extracellular matrix, focal adhesion, and extracellular vesicles (Figure 2C). In the molecular function (MF) category, enriched terms encompassed integrin binding, collagen and proteoglycan binding, enzyme regulator activity, and cell adhesion molecule binding, underscoring the roles of these genes in tumor–matrix interactions and cell signaling (Figure 2D). Kyoto Encyclopedia of Genes and Genomes (KEGG) pathway analysis revealed significant enrichment in multiple signaling cascades linked to tumor biology, including the hypoxia-inducible factor-1 signaling pathway (HIF-1), Ras-proximate-1 (Rap1), rat sarcoma viral oncogene homolog (Ras), MAPK, and focal adhesion pathways, as well as broader categories such as pathways in cancer, advanced glycation end products (AGE)–receptor for advanced glycation end products (RAGE) signaling, and cholesterol metabolism (Figure 2E). Collectively, these findings indicate that the 107 overlapping DEGs are enriched for biological processes and pathways related to angiogenesis, extracellular matrix remodeling, and cell adhesion, which are commonly implicated in osteosarcoma progression.

### 2.3. Protein–Protein Interaction (PPI) Network Construction and Identification of Hub Genes

**Figure 3:**
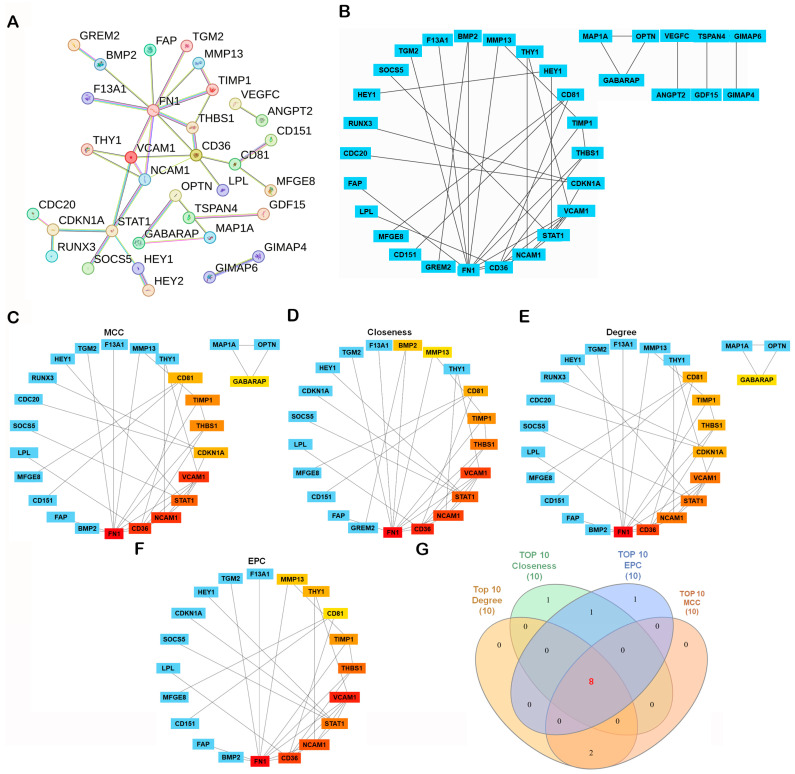
Construction and Hub Gene Analysis of the Protein–Protein Interaction (PPI) Network. (**A**) A PPI network was constructed from the 107 DEGs using the STRING database with the minimum required interaction score set to high confidence (0.700). Disconnected nodes were omitted from the visualization for clarity. PPI network of DEGs generated using the STRING database. Edges indicate interaction evidence; line color denotes the type of supporting evidence. Active interaction sources included text mining, experiments, curated databases, co-expression, neighborhood, gene fusion, and co-occurrence. (**B**–**F**) The network was visualized and analyzed in Cytoscape v3.10.2. Network modules were identified using the MCODE plugin, and key hub genes were determined with the CytoHubba plugin based on Maximal Clique Centrality (MCC), Closeness, Degree, and Edge Percolated Component (EPC) algorithms. In the CytoHubba ranking panels, warmer colors indicate higher-ranked hub genes (higher centrality), whereas cooler colors indicate lower ranks/not in the top list (please match the color meaning to the figure). (**G**) Overlapping hub genes among the four algorithms were visualized with a Venn diagram. In the Venn diagram (**G**), each colored set corresponds to the top-10 list from one algorithm (MCC, Closeness, Degree, EPC), and numbers denote overlapping genes.

**Figure 4:**
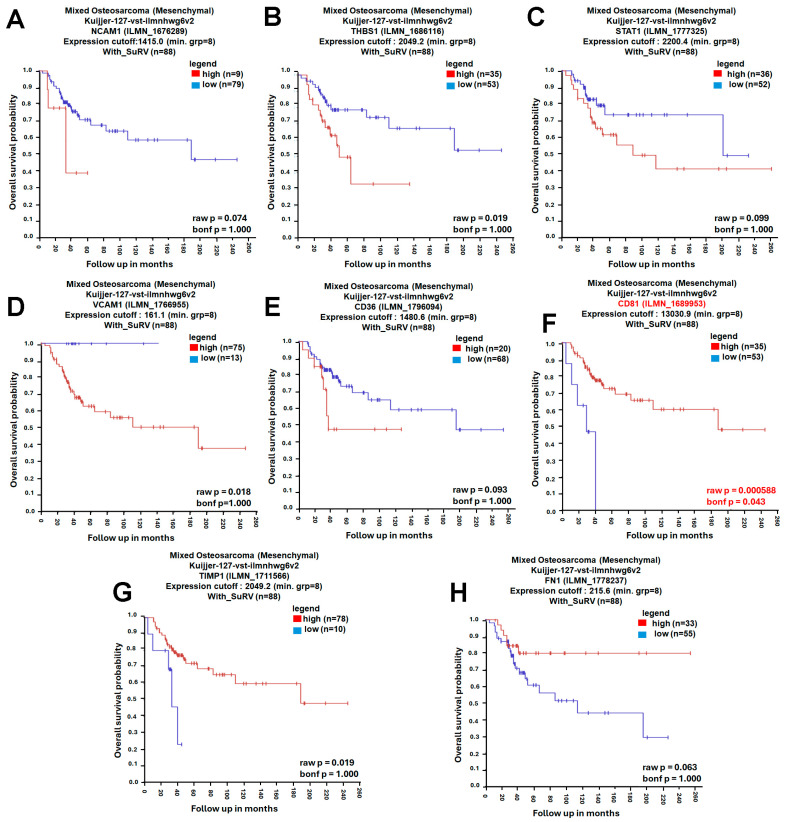
Prognostic Evaluation of Hub Genes in Osteosarcoma. (**A**) Kaplan–Meier survival curve of NCAM1; (**B**) Kaplan–Meier survival curve of THBS1; (**C**) Kaplan–Meier survival curve of STAT1; (**D**) Kaplan–Meier survival curve of VCAM1; (**E**) Kaplan–Meier survival curve of CD36; (**F**) Kaplan–Meier survival curve of CD81; (**G**) Kaplan–Meier survival curve of TIMP1; (**H**) Kaplan–Meier survival curve of FN1. The prognostic significance of these eight hub genes was analyzed using the R2 Genomics Analysis and Visualization Platform. Kaplan–Meier survival curves were generated with the KaplanScan function to compare overall survival between high- and low-expression groups. Red and blue curves indicate the high- and low-expression groups, respectively (as shown in the legend). Bonferroni-corrected *p*-values were used to determine statistical significance, and statistical significance was set at bonf *p* < 0.05.

A protein–protein interaction (PPI) network was constructed using the STRING database (https://string-db.org/, accessed on 4 November 2025), with the minimum required interaction score set to high confidence (0.700). The resulting network showed significant enrichment (PPI enrichment *p*-value < 1.0 × 10^−16^), indicating that the observed connectivity is unlikely to occur by chance (Figure 3A). Network clustering with the Molecular Complex Detection (MCODE) algorithm revealed densely connected modules representing potential functional complexes (Figure 3B). To identify the most influential genes within the network, CytoHubba in Cytoscape (v3.10.2; Cytoscape Consortium, San Diego, CA, USA) was applied using four topological algorithms—Maximal Clique Centrality (MCC), Closeness, Degree, and Edge Percolated Component (EPC)—each ranking gene by network centrality and connectivity. The top 10 hub genes from each algorithm were compared using a Venn diagram, identifying eight shared hub genes: neural cell adhesion molecule 1 (*NCAM1*), thrombospondin-1 (*THBS1*), signal transducer and activator of transcription 1 (*STAT1*), vascular cell adhesion molecule 1 (*VCAM1*), *CD36*, *CD81*, tissue inhibitor proteinases 1 (*TIMP1*), and fibronectin 1 (*FN1*) (Figure 3C–G). Subsequent survival analysis using the R2: Genomics Analysis and Visualization Platform (Amsterdam University Medical Centers/Academic Medical Center, Amsterdam, The Netherlands; https://r2.amc.nl; accessed 6 November 2025) and Visualization Platform revealed that among these candidates, only *CD81* expression was significantly associated with overall survival (OS) in osteosarcoma, whereas the other eight genes showed no significant prognostic association (Figure 4). Collectively, these analyses prioritize *CD81* as a hub gene and a candidate prognostic biomarker for further validation.

### 2.4. CD81 Expression Patterns in Osteosarcoma Tissues and Cell Lines

**Figure 5:**
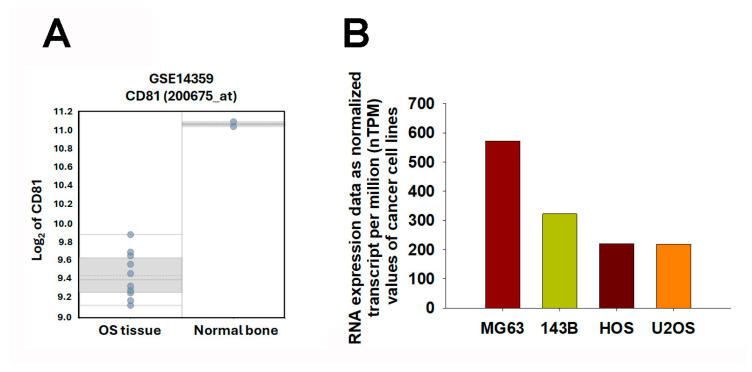
Expression Profiling of *CD81* in Osteosarcoma Tissues and Cell Lines. (**A**) *CD81* expression levels were assessed in osteosarcoma and non-malignant controls using the GSE14359 dataset. Each dot represents one sample. The box indicates the interquartile range (25th–75th percentiles), the solid line denotes the median, the dotted line denotes the mean, and the whiskers indicate the minimum and maximum values. (**B**) *CD81* expression levels were compared across osteosarcoma cell lines using the Human Protein Atlas (HPA) Cell Line Atlas. Transcriptomic data were expressed as normalized transcripts per million (nTPM) to illustrate relative *CD81* expression across cell line models. In (**B**), bars represent nTPM values for each cell line; bar colors are used only to distinguish cell lines. Statistical significance was set at *p* < 0.05.

To further explore the relationship between *CD81* expression and osteosarcoma malignancy, we analyzed transcriptomic data from both patient samples and cell lines. In the GSE14359 dataset, *CD81* expression was markedly lower in conventional osteosarcoma tissues than in the osteoblast control sample (Figure 5A). Consistently, analysis of transcriptomic data from the Human Protein Atlas (HPA) (v25.0; Stockholm, Sweden; https://www.proteinatlas.org/; accessed 6 November 2025; atlas updated 11 November 2025) revealed a similar trend among osteosarcoma cell lines. Specifically, *CD81* expression was substantially lower in commonly used osteosarcoma cell line models (e.g., 143B, HOS, and U2OS), whereas relatively higher expression levels were observed in the osteoblast-like MG63 cell line (Figure 5B). These findings collectively suggest that *CD81* expression inversely correlates with osteosarcoma aggressiveness, implying that CD81 may act as a potential tumor suppressor in osteosarcoma biology. These observations are consistent with a negative association between *CD81* expression and malignant phenotypes in osteosarcoma models, supporting *CD81* as a candidate biomarker for further validation.

### 2.5. Prediction and Identification of Hsa-miR-582-5p Targeting CD81 in Osteosarcoma

**Figure 6:**
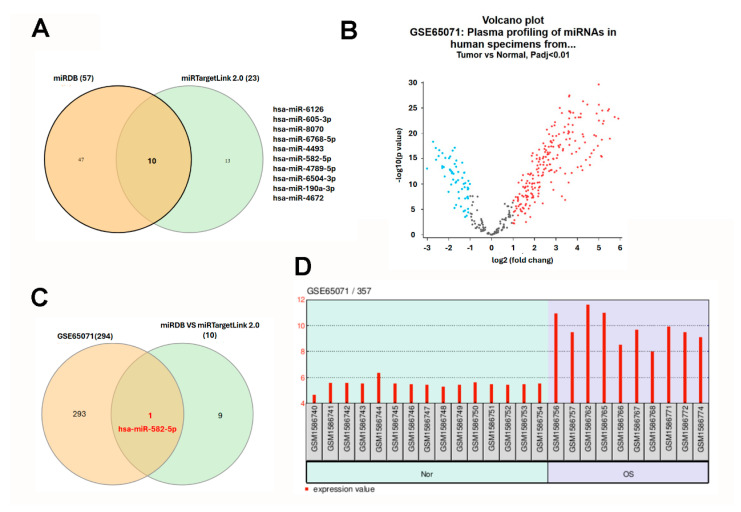
Identification of hsa-miR-582-5p as a circulating miRNA candidate linked to *CD81* target prediction in osteosarcoma. (**A**) Candidate miRNAs predicted to target *CD81* were retrieved from miRDB and miRTargetLink 2.0, and overlapping candidates were identified by Venn analysis. (**B**) Differentially expressed circulating miRNAs were analyzed in GSE65071 by comparing localized osteosarcoma plasma with healthy control plasma. For the Venn diagrams (**A**,**C**), each colored circle corresponds to one source (miRDB vs miRTargetLink 2.0; predicted vs GSE65071), and numbers indicate counts in each region. For the volcano plot (**B**), each dot represents one miRNA; red = upregulated, blue = downregulated, and gray = not significant (based on the stated threshold). (**C**) Intersection of predicted *CD81*-related miRNAs with dysregulated plasma miRNAs. (**D**) Plasma hsa-miR-582-5p levels in GSE65071. For (**D**), each bar represents one sample; background shading indicates the Normal and OS groups, and the red bars denote expression values. Statistical significance was set at adj. *p* < 0.01.

Given the pivotal role of microRNAs (miRNAs) in post-transcriptional gene regulation and their established dysregulation in cancer, we next sought to identify miRNAs that may contribute to the suppression of *CD81* in osteosarcoma. Using the miRDB database (Washington University School of Medicine, St. Louis, MO, USA; http://mirdb.org/; accessed 12 November 2025), we identified 57 candidate miRNAs predicted to target *CD81*. miRDB employs the MirTarget algorithm, which integrates high-throughput sequencing data and machine learning-based modeling to predict miRNA–mRNA interactions. In parallel, analysis through miRTargetLink 2.0 (v2.0; Saarland University, Saarbrücken, Germany; https://ccb-compute.cs.uni-saarland.de/mirtargetlink2/; accessed 12 November 2025) identified 23 miRNAs potentially targeting *CD81*, based on experimentally validated (miRTarBase, Taiwan Bioinformatics Institute Core Facility, Hsinchu, Taiwan; https://mirtarbase.cuhk.edu.cn/; accessed 12 November 2025) and predicted datasets. By intersecting both prediction results using a Venn diagram, we identified 10 overlapping miRNAs that consistently target *CD81* across platforms (Figure 6A). To determine which of these miRNAs were dysregulated in osteosarcoma, we analyzed the GSE65071 dataset, which profiles circulating miRNA expression in plasma samples from osteosarcoma patients and healthy controls. In this analysis, only localized OS plasma samples were compared with healthy controls. A total of 311 differentially expressed miRNAs were identified, including significantly upregulated (red dots) and downregulated (blue dots) species (Figure 6B). Intersection of these dysregulated miRNAs with the 10 predicted *CD81*-targeting miRNAs revealed hsa-miR-582-5p as the only consistently overlapping candidate (Figure 6C). Notably, hsa-miR-582-5p expression was significantly elevated in the plasma of osteosarcoma patients compared with healthy individuals (Figure 6D). These results nominate hsa-miR-582-5p as a circulating miRNA candidate that is upregulated in localized osteosarcoma plasma and is predicted to target *CD81*. Together with the observed downregulation of *CD81* in osteosarcoma transcriptomic datasets, these findings support a putative hsa-miR-582-5p–*CD81* relationship that warrants validation in matched clinical specimens and functional assays.

## 3. Discussion

Osteosarcoma (OS) remains a highly aggressive malignancy with limited improvement in outcomes for metastatic or recurrent disease [1,2,3,4]. In this study, we applied a multi-dataset integrative bioinformatic framework to prioritize candidate prognostic biomarkers in OS. Across three independent transcriptomic datasets, *CD81* emerged as a hub gene that was downregulated in osteosarcoma compared with osteoblast controls and whose higher expression was associated with more favorable overall survival in an external cohort. In addition, by integrating miRNA target prediction with circulating miRNA profiling from localized OS plasma samples, we nominated hsa-miR-582-5p as a candidate circulating miRNA predicted to target *CD81*, supporting a putative hsa-miR-582-5p–*CD81* relationship that warrants experimental validation.

CD81, a tetraspanin family protein, participates in multiple cellular processes including cell–cell and cell–matrix adhesion, directional migration, and signal transduction [5]. Within tetraspanin-enriched microdomains, CD81 orchestrates interactions among integrins, growth factor receptors, and cytoskeletal proteins, thereby regulating cell–matrix communication and motility [6]. In numerous malignancies—including hepatocellular carcinoma, lung cancer, and prostate cancer—dysregulated *CD81* expression has been implicated in malignant transformation, metastatic competence, and acquired therapy resistance [14,15]. Our integrative transcriptomic analysis revealed substantially reduced *CD81* expression in osteosarcoma tissues and aggressive cell lines (143B, HOS, U2OS) relative to osteoblast controls and osteoblast-like cell line models (e.g., MG63). Inverse correlation analysis demonstrated that patients harboring higher *CD81* expression achieved significantly prolonged overall survival, suggesting that higher *CD81* expression is associated with more favorable outcomes and may be consistent with a tumor-suppressive role in OS, pending validation. This finding aligns with CD81’s known role in preserving membrane integrity and constraining pathological cell migration through regulation of adhesion complexes. Collectively, these data suggest that *CD81* downregulation may facilitate osteosarcoma progression by disrupting cell–matrix adhesion and conferring enhanced invasive capacity.

Enrichment analyses of the overlapping DEGs revealed robust associations with hallmark oncogenic processes, particularly angiogenesis and metastatic dissemination. Gene ontology and pathway enrichment analyses (GO, KEGG) identified critical signaling networks including focal adhesion, vasculature development, HIF-1α, Ras, and MAPK cascades—all established modulators of osteosarcoma invasiveness and chemoresistance [16]. The enrichment of *CD81*-associated DEGs in pathways related to focal adhesion, extracellular matrix organization, and integrin-mediated signaling is consistent with pathways in which CD81 has been implicated in other contexts as a membrane-organizing scaffold that coordinates adhesion complexes and intracellular signaling cascades. Acting through pathways such as FAK, PI3K/AKT, and ERK, CD81 contributes to cytoskeletal remodeling and directional migration, processes essential for maintaining cellular architecture and motility control. Consistent with this, the loss or suppression of *CD81* expression in osteosarcoma may disrupt cell–matrix interactions, reduce adhesion stability, and facilitate invasive and metastatic behavior. Integrating these findings with the identified hsa-miR-582-5p/*CD81* putative axis suggests that dysregulated post-transcriptional control of *CD81* may enhance osteosarcoma aggressiveness through modulation of adhesion- and signaling-related networks. This refined perspective shifts the focus from vesicular trafficking to signaling imbalance at the plasma membrane, providing a more direct and experimentally tractable framework for understanding CD81-mediated regulation in osteosarcoma pathogenesis.

miRNAs function as post-transcriptional regulators critically governing osteosarcoma biology, including cellular proliferation, migration, apoptosis evasion, and chemoresistance [10,11,12,13,17]. In our comprehensive analysis, hsa-miR-582-5p emerged as the sole overlapping miRNA predicted to target *CD81* across multiple independent databases and GEO datasets. Notably, miR-582-5p expression was significantly elevated in the plasma of OS patients relative to healthy controls (GSE65071), which is consistent with a circulating miRNA signal and, together with in silico target prediction, nominates a testable hypothesis that miR-582-5p may be related to *CD81* regulation in osteosarcoma; however, circulating miRNA levels cannot be used as a surrogate for tumor-intracellular miRNA activity. The pathobiological relevance of miR-582-5p has been extensively documented across diverse malignancies, albeit with context-dependent functional outcomes. In lung adenocarcinoma, miR-582-5p suppresses *INPP5B*, a PI3K/AKT inhibitor and intrinsic tumor suppressor, thereby promoting cell proliferation and motility [18]. Prostate cancer systems biology identified miR-582-5p as a hub miRNA within metastatic signaling networks, targeting tumor suppressors including *NR3C1*, *ABHD2*, and *GSK3B* [19]. Conversely, in hepatocellular carcinoma, miR-582-5p constrains immunosuppressive signaling through *Siglec-15* downregulation, demonstrating pro-immunogenic activity [20]. In colorectal cancer, competing endogenous RNA networks revealed that lncRNAs (*UCA1*, *SNHG16*) sequester miR-582-5p, facilitating invasion and therapeutic resistance [21], while lung cancer transcriptomic profiling associated miR-582-5p with oncogenic Wnt and MAPK cascades [22]. Collectively, these cross-cancer analyses establish miR-582-5p as a pleiotropic regulator functioning through distinct molecular contexts and signaling axes.

Our integrative analyses generate a hypothesis linking elevated circulating miR-582-5p with reduced *CD81* expression observed in osteosarcoma transcriptomic datasets. Importantly, this comparison is indirect: circulating miRNAs may originate from multiple tissues and cell types and may not reflect the intracellular miRNA pool within tumor cells or the tumor microenvironment. Therefore, the proposed miR-582-5p–*CD81* relationship should be regarded as hypothesis-generating, and will require validation in matched tumor tissues (and ideally paired plasma/exosomal fractions) as well as functional assays (e.g., luciferase reporter and gain-/loss-of-function experiments). An important interpretational caveat is that the miRNA and mRNA signals were derived from different biospecimens (circulating plasma miRNAs vs. tissue transcriptomes). Therefore, elevated plasma miR-582-5p cannot be assumed to reflect the tumor-intracellular miR-582-5p pool or to indicate direct regulation of *CD81* in tumor tissues without matched tissue validation. One possibility is that circulating miR-582-5p reflects systemic tumor-associated changes, whereas tumor-intrinsic miR-582-5p–*CD81* regulation, if present, would require confirmation in matched tissue specimens. This dual behavior emphasizes the complexity of miRNA biology, where circulating and tissue-resident pools may exert distinct biological functions and prognostic significance in cancer progression. The present findings position *CD81* and hsa-miR-582-5p as candidate biomarkers for osteosarcoma diagnosis and prognosis. Accordingly, we do not claim that plasma miR-582-5p directly regulates *CD81* in osteosarcoma tissues based on the present data alone. Instead, we present circulating miR-582-5p as a non-invasive biomarker candidate and propose the miR-582-5p–*CD81* pair as a testable regulatory hypothesis to be examined in tissue- and cell-based systems.

The positive correlation between *CD81* expression and patient survival supports its potential as a prognostic biomarker for disease aggressiveness. Conversely, the upregulation of hsa-miR-582-5p in patient plasma highlights its utility as a non-invasive liquid biopsy marker for early detection. Integrating both molecules into a diagnostic framework could enhance the accuracy of existing clinical models. From a therapeutic standpoint, targeting the hsa-miR-582-5p/*CD81* axis may offer new opportunities for precision oncology. These findings may inform future studies evaluating whether modulation of the proposed relationship has therapeutic relevance; however, any translational applications require rigorous experimental validation and careful safety assessment given the context-dependent functions of CD81. Nevertheless, therapeutic manipulation requires caution: as CD81 functions vary across tissue types, systemic targeting could disrupt normal physiological communication in immune and hematopoietic systems [14,15,23].

This study is limited by its reliance on public transcriptomic datasets, which may contain platform-related variability and limited clinical annotations. Some of the GEO datasets included in this study contained a limited number of biological control samples, which constrains statistical power at the level of individual dataset comparisons. Although differential expression analyses were conducted using GEO2R with limma-based moderated t-statistics and empirical Bayes variance estimation—an approach that improves stability and is appropriate for exploratory transcriptomic analyses—results derived from any single dataset should not be interpreted as definitive. Accordingly, the analytical strategy of this study emphasized cross-dataset reproducibility rather than reliance on individual comparisons. Only genes consistently identified across multiple independent datasets, platforms, and biological contexts were retained for downstream network, survival, and miRNA analyses. This integrative design reduces dataset-specific bias and supports the interpretation of the findings as hypothesis-generating rather than confirmatory. Although bioinformatics analyses predict a functional interaction between hsa-miR-582-5p and *CD81*, experimental validation—such as luciferase reporter assays, miRNA mimic/inhibitor transfection, and *CD81* rescue experiments—is essential to confirm direct binding and causal effects. Further, mechanistic exploration of downstream signaling (e.g., PI3K/AKT, Wnt, focal adhesion) could clarify how this axis influences proliferation, migration, and exosome biogenesis. Future investigations should integrate multi-omics analyses and functional assays to validate the prognostic and mechanistic significance of the hsa-miR-582-5p/*CD81* axis. Evaluating circulating exosomal CD81 and plasma miR-582-5p in longitudinal patient cohorts could establish their roles in metastasis and therapeutic response monitoring. Collectively, these studies would help translate the bioinformatics findings into clinically applicable diagnostic and therapeutic strategies.

## 4. Materials and Methods

### 4.1. Data Acquisition and Dataset Selection

**Figure 7:**
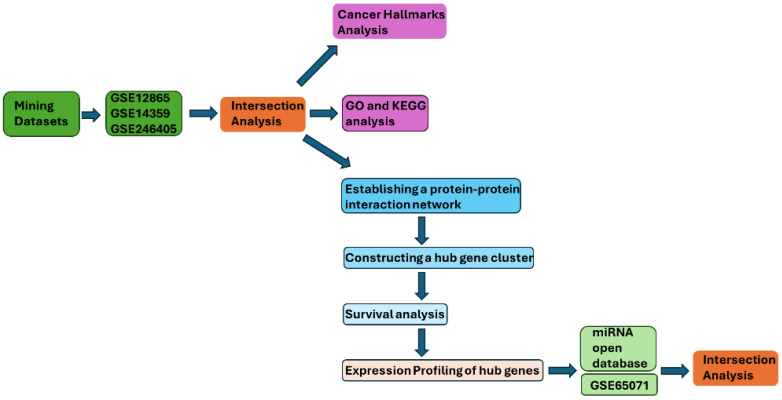
The target gene identification flowchart of this study. The flowchart summarizes the study workflow from dataset mining to DEG intersection, enrichment analyses, PPI/hub identification, survival analysis, and miRNA intersection.

Publicly available osteosarcoma-related gene expression datasets were systematically screened in the GEO database (National Center for Biotechnology Information, Bethesda, MD, USA; https://www.ncbi.nlm.nih.gov/geo/; accessed 2 November 2025). Datasets were considered eligible if they met the following criteria: (i) inclusion of osteosarcoma samples with clearly defined non-malignant bone-related controls; (ii) availability of processed expression data suitable for differential expression analysis; (iii) sufficient annotation to distinguish biological samples from technical replicates; and (iv) relevance to primary osteosarcoma biology rather than treatment response or unrelated experimental perturbations. Based on these criteria, three mRNA expression datasets—GSE12865, GSE14359, and GSE246405—were selected for integrative transcriptomic analysis. Other osteosarcoma-related GEO datasets were excluded if they lacked appropriate non-malignant controls, focused exclusively on drug-treated conditions, had insufficient sample annotation, or were not suitable for cross-dataset comparison. GSE12865 (last updated 26 July 2018) was generated using the Affymetrix Human Gene 1.0 ST Array (GPL6244) and includes six osteosarcoma tumor samples and one normal human osteoblast (HOB) sample, each profiled in technical duplicate. GSE14359 (GPL96; last updated 10 August 2018) contains five conventional primary osteosarcoma tissue samples, four osteosarcoma lung metastasis samples, and non-neoplastic primary osteoblast cells (HOBc), all profiled in technical duplicate. For differential expression analysis, only conventional primary osteosarcoma samples and HOBc controls were included, while metastatic samples were excluded to reduce biological heterogeneity. GSE246405 (last updated 17 April 2024), generated using the NextSeq 2000 platform (GPL30173), comprises four osteosarcoma RNA-seq models (NRHOS, OSKG, OSRH20115, and I063021) and one osteoblast control model (OB). Three osteosarcoma models and the osteoblast control were available with two replicates, whereas one osteosarcoma model (I063021) had a single replicate. For circulating miRNA analysis, GSE65071 (GPL19631; last updated 25 April 2016) was selected because it includes plasma samples from osteosarcoma patients and healthy controls profiled using a standardized qPCR-based platform. In the present study, 15 healthy control plasma samples and 10 localized osteosarcoma plasma samples were analyzed, while metastatic plasma samples were not included (Figure 7). Together, these datasets were selected to enable cross-dataset validation of consistently dysregulated genes and miRNAs while balancing data quality, biological relevance, and platform compatibility.

### 4.2. Identification of DEGs

DEGs between osteosarcoma and non-malignant controls were identified using the GEO2R web tool (Bethesda, MD, USA; https://www.ncbi.nlm.nih.gov/geo/geo2r/; accessed 2 November 2025), which performs differential expression analysis in R (v4.2.2) using Biobase (v2.58.0), GEOquery (v2.66.0), and limma (v3.54.0) [24]. Gene expression data from each dataset were normalized and statistically analyzed to compare tumor and control samples. DEGs were screened using a Benjamini–Hochberg adjusted *p*-value < 0.01 and an absolute log_2_ fold change (|log_2_FC|) > 1.0 as the significance thresholds. Subsequently, overlapping DEGs among the three datasets were determined using InteractiVenn (University of São Paulo, São Carlos, SP, Brazil; https://www.interactivenn.net/; accessed 3 November 2025) to obtain a robust set of consistently dysregulated genes across studies [25].

### 4.3. Hallmark Enrichment Analysis

To investigate the biological relevance of the overlapping DEGs identified from the three GEO datasets (GSE12865, GSE14359, and GSE246405), hallmark enrichment analysis was performed using the Cancer Hallmarks Analytics Tool (Semmelweis University, Budapest, Hungary; https://cancerhallmarks.com; accessed 3 November 2025). This platform maps input genes to ten predefined cancer hallmark categories through a hypergeometric statistical model based on a curated reference dataset [26]. DEGs were uploaded using official gene symbols, and enrichment significance was determined according to the adjusted *p*-value calculated by the platform. The results were visualized in a Hallmark Enrichment Plot consisting of five concentric layers. The outer ring labels the ten hallmark categories, where the size of each slice represents the number of DEGs associated with that hallmark and the color corresponds to the specific hallmark type. Each ring within the plot denotes a different adjusted *p*-value interval (1–0.0001); when the adjusted *p*-value exceeds 0.05, the corresponding slice is displayed in gray, indicating a lack of statistical significance. This visualization provided a comprehensive overview of the hallmark pathways most closely associated with osteosarcoma-related gene dysregulation.

### 4.4. Gene Ontology (GO) and Kyoto Encyclopedia of Genes and Genomes (KEGG) Pathway Analysis

Functional enrichment of the identified DEGs was carried out using ShinyGO v0.82 (South Dakota State University, Brookings, SD, USA; http://bioinformatics.sdstate.edu/go/; accessed 4 November 2025). This analysis incorporated both GO and KEGG databases to systematically classify DEGs according to their associated BPs, MFs, and CCs, as well as to identify significantly enriched signaling pathways [27,28,29]. Enrichment significance was determined using a *p*-value < 0.01 and a false discovery rate (FDR) < 0.05 as the cutoff criteria. ShinyGO ranks enrichment terms based on their FDR values, gene counts, and fold enrichment scores, providing insight into the cellular processes and molecular pathways most affected by gene dysregulation in osteosarcoma. These analyses collectively facilitated the identification of key biological mechanisms potentially contributing to tumor development and progression.

### 4.5. Protein–Protein Interaction (PPI) Network Construction and Hub Gene Identification

To explore the functional associations among the identified DEGs, a PPI network was constructed using the STRING database (University of Zurich, Lausanne, Switzerland; https://string-db.org/; accessed 4 November 2025). Interactions with a combined confidence score greater than 0.7 were considered significant and included in the analysis [30,31]. For visualization purposes, disconnected nodes were hidden in STRING to improve network readability. The resulting interaction data were imported into Cytoscape (v3.10.2; Cytoscape Consortium, San Diego, CA, USA) for network visualization and topological analysis [32,33]. Within Cytoscape, the CytoHubba plugin was applied to evaluate node connectivity and identify the most influential hub genes using four established topological algorithms: MCC, Closeness, Degree, and EPC. The MCODE plugin (version 1.5.1) was used to detect densely connected submodules under the parameters: Degree Cutoff = 2, Node Score Cutoff = 0.2, K-Core = 2, and Max Depth = 100 [32,33]. Genes consistently ranked highly across the four CytoHubba algorithms were considered key nodes within the network. Based on this integrative approach, eight hub genes—*NCAM1*, *THBS1*, *STAT1*, *VCAM1*, *CD36*, *CD81*, *TIMP1*, and *FN1*—were identified for subsequent validation and prognostic assessment.

### 4.6. Survival Analysis of Hub Genes

To assess the prognostic value of the eight hub genes (*NCAM1*, *THBS1*, *STAT1*, *VCAM1*, *CD36*, *CD81*, *TIMP1*, *FN1*, and *THY1*) in osteosarcoma, survival analyses were performed using the R2 Genomics Analysis and Visualization Platform (Amsterdam University Medical Centers/Academic Medical Center, Amsterdam, The Netherlands; https://r2.amc.nl; accessed 6 November 2025). The R2 platform is an open-access web-based environment designed to enable biomedical researchers to analyze, visualize, and interpret transcriptomic data through an intuitive, no-code interface. Within R2, the KaplanScan function was applied to evaluate the association between gene expression and overall survival in osteosarcoma patient cohorts. The KaplanScanner tool determines the optimal expression cutoff to stratify patients into high- and low-expression groups based on the log-rank test. Statistical significance was assessed using both the raw *p*-value (unadjusted) and the Bonferroni-corrected *p*-value (bonf *p*), the latter representing post hoc correction for multiple testing. Only associations meeting the Bonferroni-adjusted significance threshold were considered statistically significant.

### 4.7. CD81 Expression Profiling in Osteosarcoma Cell Lines

The Human Protein Atlas (HPA) (v25.0; Stockholm, Sweden; https://www.proteinatlas.org/; accessed 6 November 2025; atlas updated 11 November 2025) was utilized to examine the expression profile of *CD81* across osteosarcoma cell lines. The HPA is an open-access resource integrating antibody-based imaging, mass spectrometry, transcriptomics, and systems biology to systematically map protein-coding genes in human tissues, organs, and cell lines. Within its Cell Line Atlas, the HPA provides genome-wide transcriptomic data expressed as normalized transcripts per million (nTPM) values for over 1,200 human cell lines, including osteosarcoma-derived models. In this study, *CD81* nTPM values were retrieved to compare relative expression levels among osteosarcoma cell lines, thereby complementing transcriptomic analyses and providing an independent reference for *CD81* expression at the cellular level.

### 4.8. Statistical Analysis

All statistical analyses were conducted using the integrated functions provided by the aforementioned bioinformatics platforms. Differential expression analyses were performed using GEO2R, which implements the limma package and applies moderated t-statistics with empirical Bayes variance estimation. This framework is specifically designed for high-dimensional transcriptomic data and improves variance estimation by borrowing information across genes, thereby allowing exploratory inference even under conditions of limited sample size or unequal group distributions. Importantly, the use of limma does not eliminate the constraints imposed by small biological control cohorts; rather, it enables statistically principled screening of candidate genes at the exploratory stage. Multiple-testing correction was applied using the Benjamini–Hochberg method as implemented by the platform. Survival analyses were conducted using the R2 Genomics Analysis and Visualization Platform with log-rank testing and the KaplanScan procedure, with multiple-testing correction applied as reported by the platform. Unless otherwise specified, statistical significance was defined as *p* < 0.05. Where summary statistics were reported, values are presented as mean ± standard deviation (SD).

## 5. Conclusions

In summary, this multi-dataset in silico study prioritized *CD81* as a candidate prognostic biomarker in osteosarcoma and nominated a putative hsa-miR-582-5p–*CD81* relationship based on cross-dataset transcriptomic evidence, survival association, circulating miRNA profiling, and target prediction.

## Figures and Tables

**Table 1:** Summary of sample size for each dataset. Sample sizes represent biological samples or models; technical replicates were not counted as independent samples.

GSE No.	Control	Osteosarcoma	Notes
GSE12865	1	6	Osteosarcoma tumor tissues vs. normal human osteoblasts (HOBs); each biological sample profiled in technical duplicate
GSE14359	1	5	Conventional primary osteosarcoma tissues vs. non-neoplastic primary HOB; lung metastasis samples excluded
GSE246405	1	4	Osteosarcoma RNA-seq models vs. osteoblast control model; one OS model lacked a replicate

## Data Availability

All data generated or analyzed during this study are available through publicly accessible databases and bioinformatics platforms with no access restrictions. Four publicly available expression datasets (GSE12865, GSE14359, GSE246405, and GSE65071) were retrieved from the Gene Expression Omnibus (GEO) database (https://www.ncbi.nlm.nih.gov/geo/; accessed 2 November 2025)) to identify differentially expressed genes (DEGs) and circulating miRNAs associated with osteosarcoma. GSE12865 (GPL6244; Affymetrix Human Gene 1.0 ST Array; last updated 26 July 2018) includes 6 osteosarcoma tumor samples and 1 normal human osteoblast (HOB) control sample, each profiled in duplicate. GSE14359 (GPL96; Affymetrix Human Genome U133A Array; last updated 10 August 2018) contains 5 conventional primary osteosarcoma tissue samples and 4 osteosarcoma lung metastasis samples, together with non-neoplastic primary osteoblast cells (HOBc), all profiled in duplicate; for DEG identification, we focused on conventional primary osteosarcoma tissues versus HOBc controls and excluded metastatic samples to reduce biological heterogeneity. For GSE246405 (GPL30173; NextSeq 2000 RNA-seq; last updated 17 April 2024), we analyzed four osteosarcoma RNA-seq models (NRHOS, OSKG, OSRH20115, and I063021) and one osteoblast control model (OB). Three osteosarcoma models and the osteoblast control had two replicates (R1/R2), whereas one osteosarcoma model (I063021) had only a single replicate available in our analysis. GSE65071 (GPL19631; Exiqon Human V3 microRNA PCR panels I + II; last updated 25 April 2016) profiles circulating miRNAs in plasma specimens; in the present study, we analyzed 15 healthy control plasma samples and 10 localized osteosarcoma plasma samples, while metastatic osteosarcoma plasma samples (n = 10) available in the series were not included. These datasets were selected based on sample adequacy, the availability of osteosarcoma and appropriate control groups, and the use of standardized high-quality platforms. The integration of these datasets provided a foundation for cross-dataset comparison and nomination of robust candidate biomarkers. All raw and processed data files are publicly available through GEO. Additionally, all bioinformatics tools and databases utilized in this study are freely accessible online.

## Abbreviations

The following abbreviations are used in this manuscript:

Akt	Protein kinase B (PKB)
BP	Biological Process
CC	Cellular Component
CD81	Cluster of Differentiation 81
DEG(s)	Differentially Expressed Gene(s)
ECM	Extracellular Matrix
EMT	Epithelial–Mesenchymal Transition
EPC	Edge Percolated Component
ERK	Extracellular signal-regulated kinase
FAK	Focal Adhesion Kinase
FDR	False Discovery Rate
FN1	Fibronectin 1
GEO	Gene Expression Omnibus
GO	Gene Ontology
GPL	GEO Platform accession (microarray/seq platform ID)
GSE	GEO Series accession (dataset ID)
HIF-1	Hypoxia-Inducible Factor-1 signaling pathway
HPA	Human Protein Atlas
ICAM1	Intercellular Adhesion Molecule 1
KEGG	Kyoto Encyclopedia of Genes and Genomes
KM	Kaplan–Meier
MCC	Maximal Clique Centrality
MF	Molecular Function
miRNA	MicroRNA
MNC	Maximum Neighborhood Component
MCODE	Molecular Complex Detection
NCAM1	Neural Cell Adhesion Molecule 1
nTPM	Normalized Transcripts Per Million
OS	Osteosarcoma
PPI	Protein–Protein Interaction
Rap1	Ras-proximate-1
Ras	Rat Sarcoma Viral Oncogene Homolog
SD	Standard Deviation
STAT1	Signal Transducer and Activator of Transcription 1
TEM(s)	Tetraspanin-Enriched Microdomain(s)
THBS1	Thrombospondin 1
THY-1	Thy-1 Cell Surface Antigen
TIMP1	Tissue Inhibitor protease 1
VCAM1	Vascular Cell Adhesion Molecule 1
